# Quantum Sensing of Free Radical Generation in Mitochondria of Single Heart Muscle Cells during Hypoxia and Reoxygenation

**DOI:** 10.1021/acsnano.3c07959

**Published:** 2024-01-18

**Authors:** Siyu Fan, Han Gao, Yue Zhang, Linyan Nie, Raquel Bártolo, Reinier Bron, Hélder A. Santos, Romana Schirhagl

**Affiliations:** † Department of Biomaterials and Biomedical Technology, University Medical Center Groningen, University of Groningen, 9713 AV Groningen, The Netherlands; ‡ Drug Research Program, Division of Pharmaceutical Chemistry and Technology, Faculty of Pharmacy, University of Helsinki, FI-00014, Helsinki, Finland

**Keywords:** diamonds, nanodiamonds, quantum sensing, NV centers, hypoxia

## Abstract

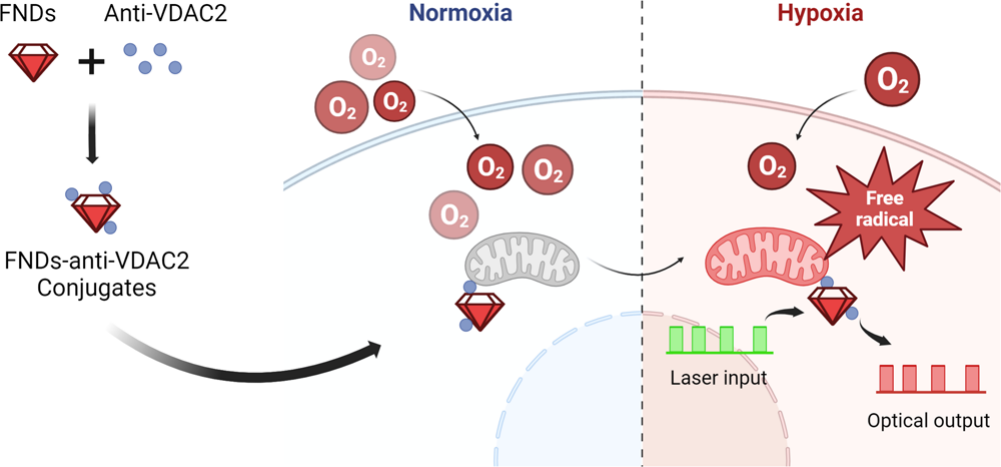

Cells are damaged during hypoxia (blood supply deprivation) and reoxygenation (oxygen return). This damage occurs in conditions such as cardiovascular diseases, cancer, and organ transplantation, potentially harming the tissue and organs. The role of free radicals in cellular metabolic reprogramming under hypoxia is under debate, but their measurement is challenging due to their short lifespan and limited diffusion range. In this study, we employed a quantum sensing technique to measure the real-time production of free radicals at the subcellular level. We utilize fluorescent nanodiamonds (FNDs) that exhibit changes in their optical properties based on the surrounding magnetic noise. This way, we were able to detect the presence of free radicals. To specifically monitor radical generation near mitochondria, we coated the FNDs with an antibody targeting voltage-dependent anion channel 2 (anti-VDAC2), which is located in the outer membrane of mitochondria. We observed a significant increase in the radical load on the mitochondrial membrane when cells were exposed to hypoxia. Subsequently, during reoxygenation, the levels of radicals gradually decreased back to the normoxia state. Overall, by applying a quantum sensing technique, the connections among hypoxia, free radicals, and the cellular redox status has been revealed.

## Introduction

Free radicals play essential roles in cellular responses, acting as secondary messengers within controlled levels.^1^ However, high concentrations of free radicals can lead to oxidative stress and cytotoxicity by oxidizing nucleic acids, proteins, and lipids, particularly in mitochondria,^2^ where radicals are mainly produced at redox centers of the respiratory chain.^3^

Hypoxia, characterized by low oxygen levels, occurs in various physiological and pathological conditions, including ischemic disorders, atherosclerosis, and cancer.^3,4^ Hypoxia induces the stabilization of the subunit α of the hypoxia-inducible factor 1 (HIF-1α), triggering a cellular adaptive response mediated by HIF-1, which regulates transcription under hypoxia.^3,4^ Mitochondria are major targets of this process, as HIF-1α can inhibit pyruvate dehydrogenase (PDH) activity, limiting substrates for oxidative phosphorylation (OXPHOS).^5^

The role of free radicals in cell adaptation to hypoxia remains debated,^3^ with some studies indicating increased radical production (mostly derived from the mitochondrial electron transport chain (ETC))^6,7^ and others suggesting the opposite.^8,9^ Though the discovery of HIF-1α by Semenza et al.^10^ offered a molecular basis for determining the mechanism of responses to oxygen deprivation, the cellular and molecular biology of hypoxia is not fully understood yet.

To investigate the connections among hypoxia, free radical generation, and cellular redox status, we utilized H9c2 myoblasts, a subclone from embryonic BD1X rat heart tissue, as a representative for cardiomyocytes. Some molecules, like mitochondrial matrix-targeted superoxide indicator^8^ and free-radical generator,^11^ were developed to measure free radicals. However, all of these fluorescent molecules suffer from photobleaching. As a result, they reveal the history of the sample instead of its current state. Here we were interested in levels of free radicals in H9c2 cells adapted to decreased O_2_ concentration (<1% O_2_) near mitochondria in real time. We employed nonbleaching fluorescent nanodiamonds^12^ (FNDs) coated with VDAC2 antibody targeted to mitochondria. These FNDs contained nitrogen-vacancy (NV) centers capable of sensing the free electrons of radicals at the nanoscale. NV center based sensing has already been applied for several applications in physics including the sensing of magnetic nanostructures, nanoparticles, paramagnetic ions, or spin defects.^13−17^ NV centers in diamonds offer the capability to perform measurements under extreme pressures or temperatures.^18,19^ Their potential in biology has already been demonstrated, such as visualizing spin labels in fixed cell slices^20^ and measuring iron-containing protein,^21^ as well as enabling nanoscale temperature measurements^18,22^ and orientation measurements,^23,24^ depending on the specific measurement mode. Recently, our research group has demonstrated the detection of free radical generation at the nanoscale with this method. Since then, this method has been applied to various biological systems, including aging yeast cells,^25^ immune cells,^26,27^ and endothelial cells,^28^ during viral infection^29^ or during sperm cell maturation.^30^

**Figure 1:**
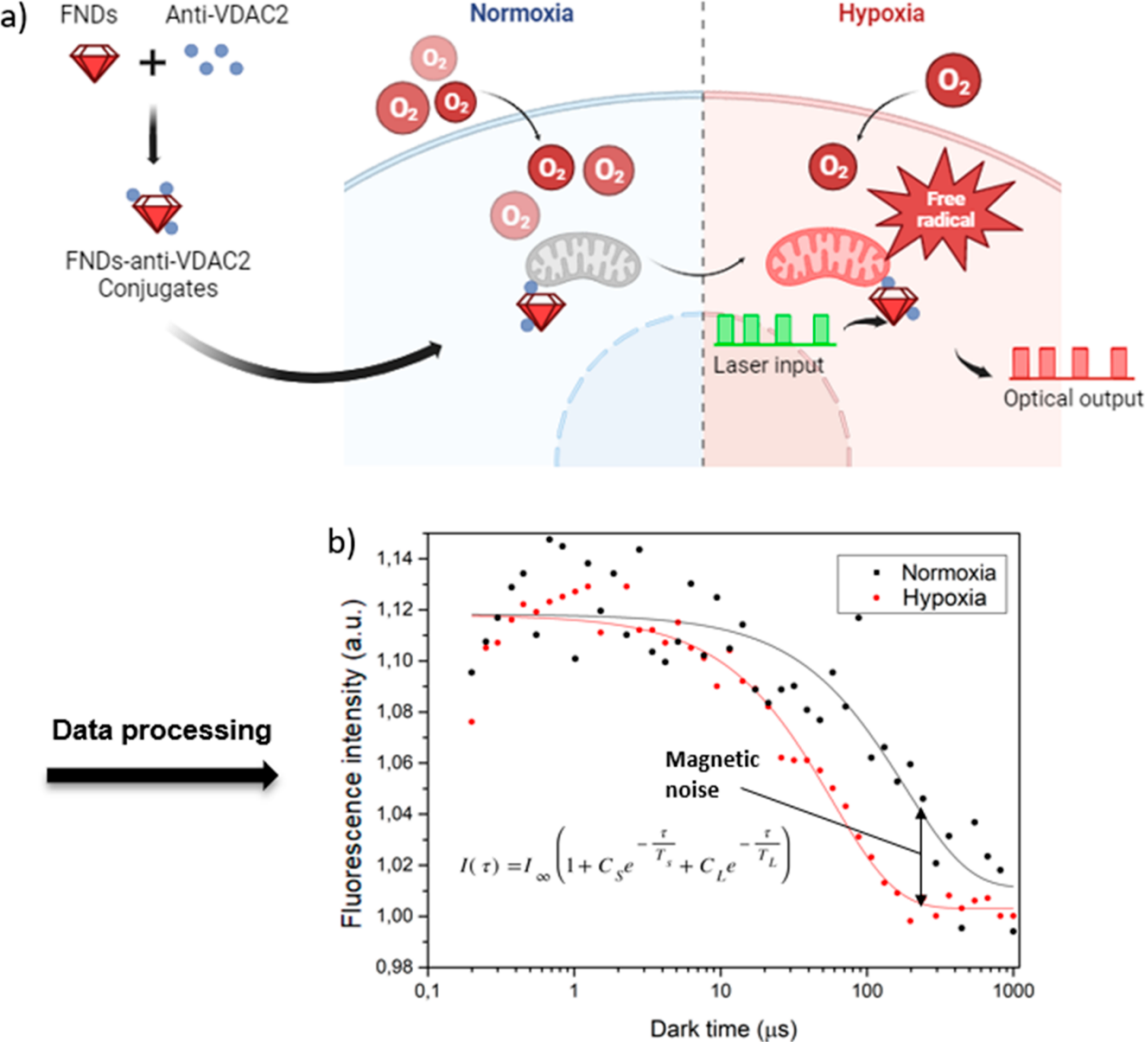
Schematic representation of the experiments in this article. Nanodiamonds are targeted to mitochondria and sense surrounding free radical generation in heart muscle cells (H9c2 myoblasts). (a) The green blocks (561 nm) represent laser pulses, while the red blocks show the photoluminescence (PL) from the NV centers. The signal detected during these pulses is used to create the T1 relaxation curve. (b) To this end, different dark times are plotted against the PL intensity at the respective dark times to generate a T1 curve. The time to reach equilibrium (T1) is reduced in the presence of free radicals. The red and black lines show T1 measured at normoxia and hypoxia with nanodiamonds targeted to the mitochondria. To ensure accuracy, each pulsing sequence was performed 10,000 times, ensuring a good signal-to-noise ratio.

Here we show the detection of free radicals near mitochondria during hypoxia and reoxygenation in H9c2 cells, providing insights into hypoxia-related mechanisms, as illustrated in Figure 1.

## Results and Discussions

### Characterization of the Materials

**Figure 2:**
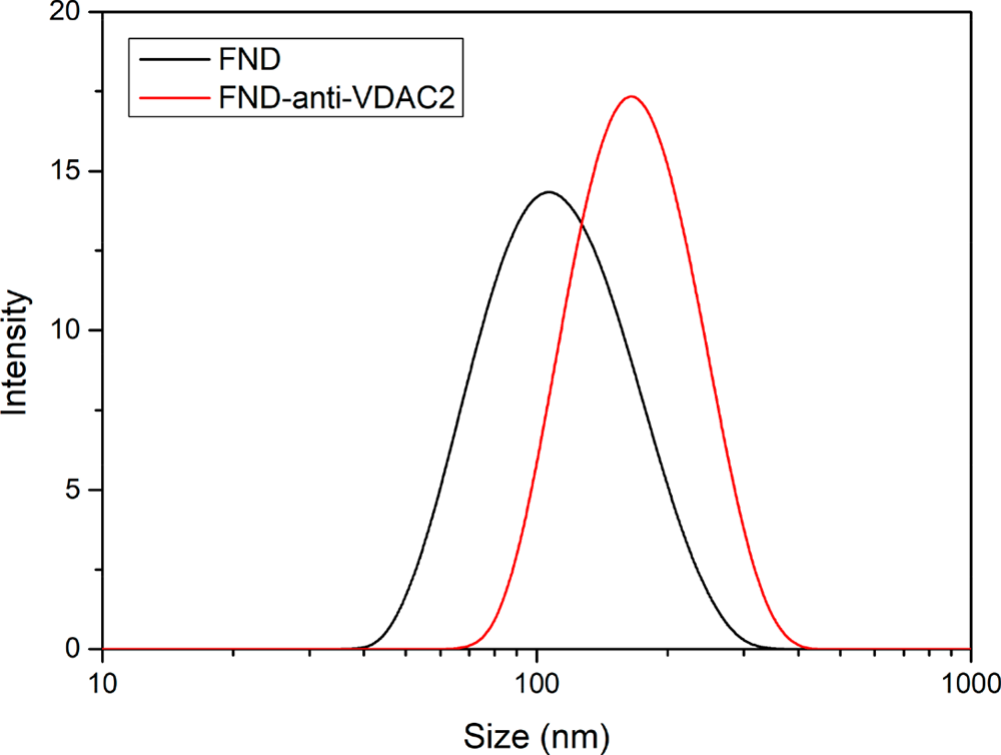
Size distribution of bare FNDs and FND-anti-VDAC2. The hydrodynamic diameter was determined by measuring dynamic light scattering using a Malvern ZetaSizer Nano system.

As shown in Figure 2, for uncoated FND (black), the size was 102 nm (polydispersity index (PDI) = 0.171). FND-anti-VDAC2 (red) was 182 nm in size (PDI = 0.176). The increase in the particle size indicated that bare FNDs were successfully coated by antibodies and aggregated slightly. A similar result was also demonstrated in previous work.^26^

### Impact of Hypoxia

**Figure 3:**
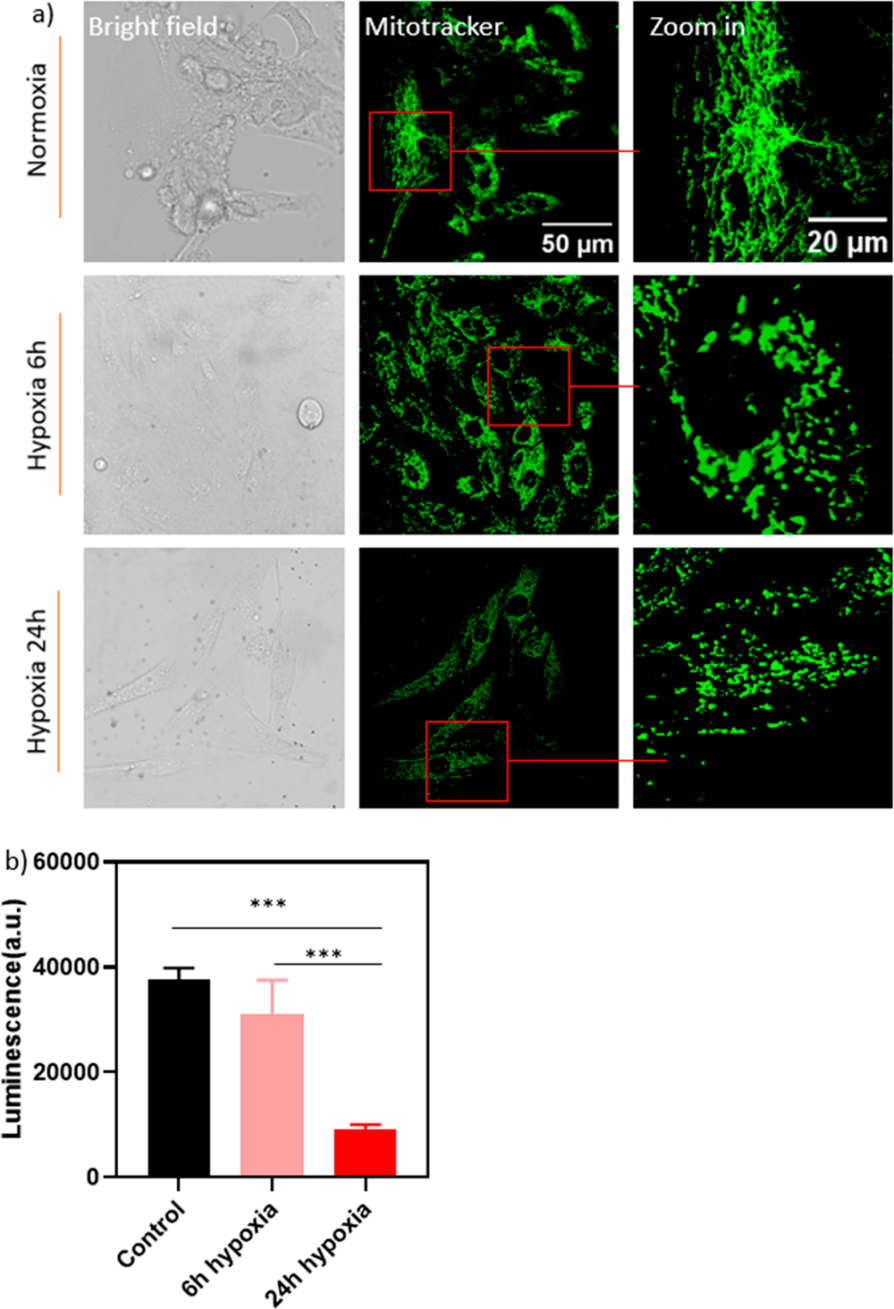
Impact of hypoxic stress on H9c2 cells. (a) Representative Z-stack confocal microscopy images were taken before and after different hypoxic treatments (<1% oxygen). (b) Cell viability test after hypoxia treatment by a cell titer assay. Mitotracker Green staining is shown in green. Error bars represent standard deviations. Statistical significance was evaluated by using one-way ANOVA, and statistical differences are indicated by ***p ≤ 0.001.

We proceeded to investigate the susceptibility of H9c2 cells to hypoxia and the resulting metabolic stress by examining the mitochondrial morphology (Figure 3a) and determining the cell viability (Figure 3b).

Mitochondria exhibit a dynamic tubular network, which undergoes frequent fission and fusion events regulated by metabolic changes within the cell.^3^ After cells were exposed to hypoxia (see Figure 3a), a clear alteration in mitochondrial morphology was observed from a tubular network to a particle-shaped structure, while the overall cell morphology remained relatively unchanged. Impairment of mitochondrial fusion is associated with mitochondrial depolarization and disrupted mitochondrial distribution within cells.^46^

Furthermore, prolonged exposure of H9c2 cells to hypoxic conditions significantly affected the ATP levels (Figure 3b). Decreased oxygen availability leads to reduced glucose oxidation, prompting cells to rely on anaerobic glycolysis for ATP production, resulting in lactate accumulation (Pasteur effect).^47^ Consequently, a decline in the ATP levels was expected under hypoxic conditions. The cells exposed to hypoxia displayed distinct time-dependent variations in ATP levels (Figure 3b). Cell viability of H9c2 cells was reduced by 16% and significantly 75% (*p* ≤ 0.001) at 6 and 24 h after hypoxia, respectively.

**Figure 4:**
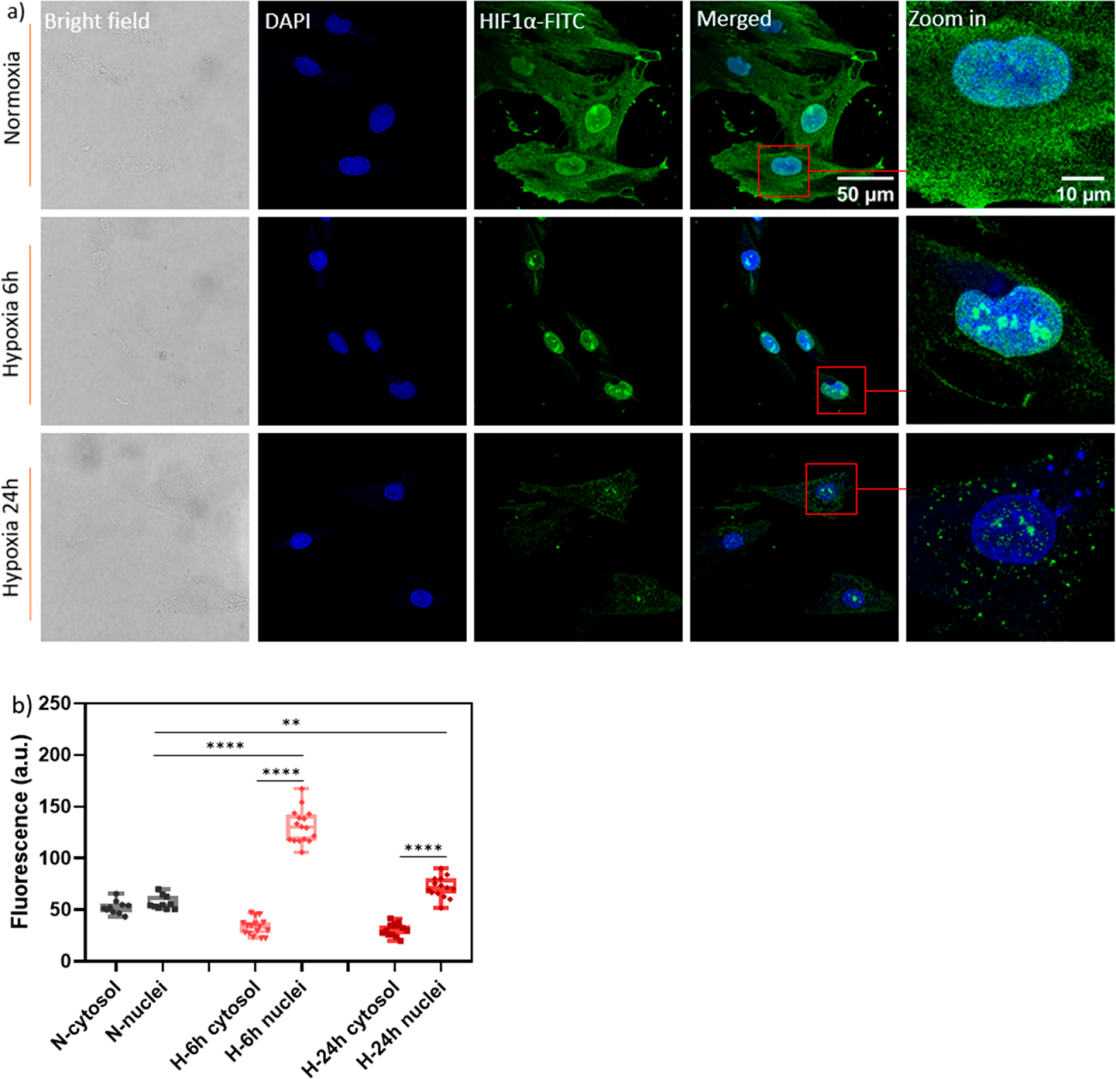
Assessment of HIF-1α expression and nuclei translocation in H9c2 cells under different hypoxic conditions. (a) Representative Z-stack confocal microscopy images taken before and after different hypoxic treatments (<1% oxygen). Cells were incubated with the anti-HIF-1α antibody followed by a FITC-conjugated secondary antibody. (b) Mean optical intensity in different regions of interest were anaylzed using FIJI. N = normoxia; H = hypoxia. Color code in (a): green, FITC (for HIF-1α); blue, DAPI. Whiskers represent min and max values; data between each group were analyzed using a two-way ANOVA analysis: **p ≤ 0.01, ****p ≤ 0.0001.

In order to investigate cellular responses under various hypoxic conditions, we examined the expression level and intracellular localization of HIF1α, which is a key regulator for adapting to low oxygen environments.^10^ Under normoxia, HIF1α was primarily found in the cytosol (Figure 4a). However, during hypoxia, HIF1α tended to translocate to the nucleus. This translocation occurs when oxygen levels fall below a critical threshold, leading to reduced activity of the HIF prolyl hydroxylases (HIF PHDs) that depend on oxygen for their function.^10^ As a result, the stabilization of HIF1α occurs, allowing it to enter the nucleus. Conversely, under normoxia, HIF1α is continuously hydroxylated, leading to its degradation by proteasomes.^48^

The quantification of HIF1α levels is shown in Figure 4b. Compared with the relatively equal distribution between the nucleus and cytoplasm under normoxia, a significant translocation of HIF1α was observed after both 6 and 24 h of hypoxic treatment. Additionally, after exposure to different durations of hypoxia, HIF1α exhibited higher expression levels in the nucleus. Interestingly, a lower accumulation of HIF1α in the nucleus was observed at 24 h compared to 6 h, suggesting a potential cellular response overload.^49^

### FND Uptake

**Figure 5:**
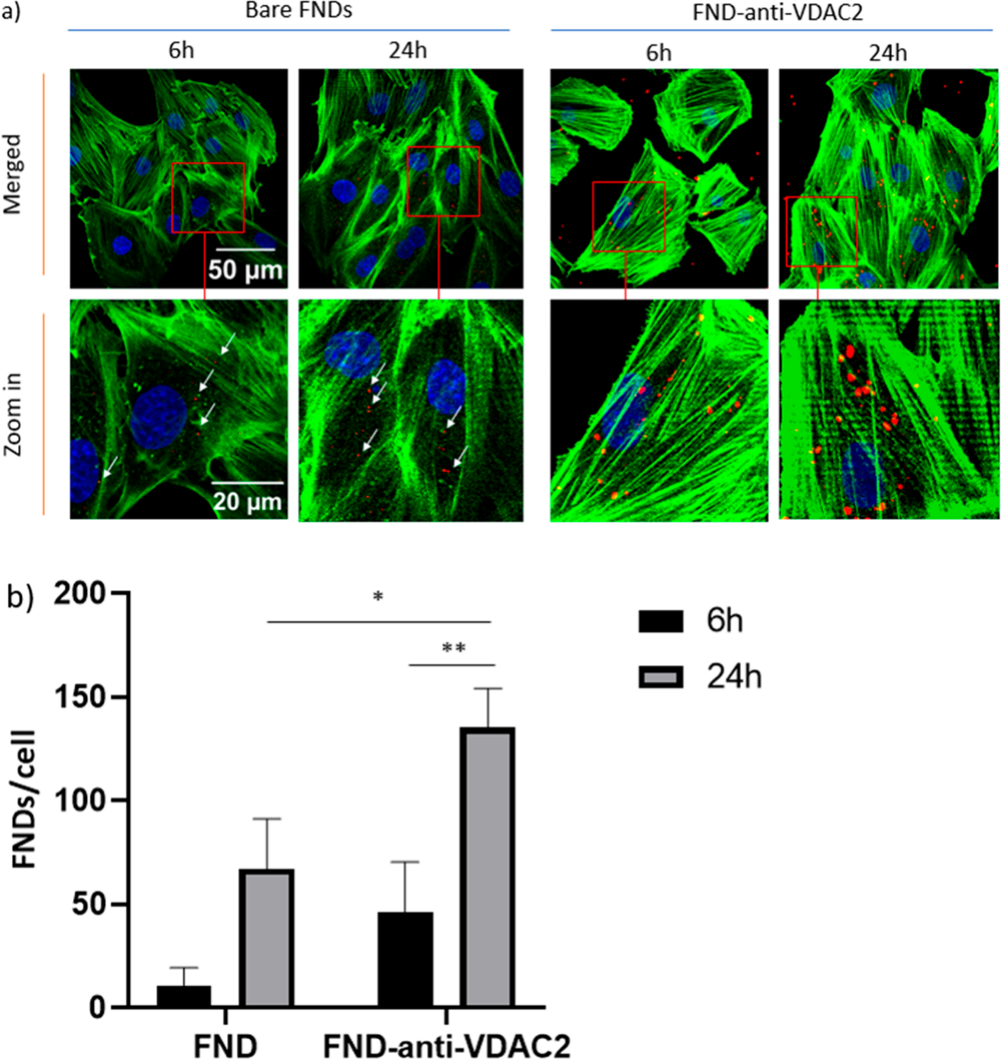
FND uptake by H9c2 cells: (a) H9c2 cells were incubated with 1 μg/mL FNDs or FND-anti-VDAC2 for 6 or 24 h. Color code: green, phalloidin-FITC, staining actin filaments; blue, DAPI (staining DNA); red, FNDs(-anti-VDAC2). (b) Quantified FND uptake per cell after incubating for 6 or 24 h. The experiment was performed in independent triplicates. In each experiment, 60 cells were randomly selected per group. Error bars represent standard deviations. Significant differences between groups were evaluated by using a two-way ANOVA, and the statistical difference is indicated by *p ≤ 0.05 and **p ≤ 0.01.

To perform relaxometry experiments near mitochondria, diamond particles must be internalized by the cells. As shown in Figure 5a, H9c2 cells ingested diamond particles and exhibited different uptake abilities (Figure 5b).

In H9c2 cells, particle uptake showed a time-dependent increase for both types of FNDs (Figure 5b). Here it is beneficial that we used FNDs with ensembles of NV centers, which are easier to detect. At 24 h, compared to bare FNDs, the number of ingested particles significantly increased for the FND-anti-VDAC2 group, indicating particles coated with antibodies might be easier to endocytose. It has been reported that VDAC2 can promote clathrin-independent endocytosis,^50^ which may be related to the increased endocytosis of anti-VDAC2-coated FNDs.

### Colocalization of Mitochondria and FND/FND-anti-VDAC2

**Figure 6:**
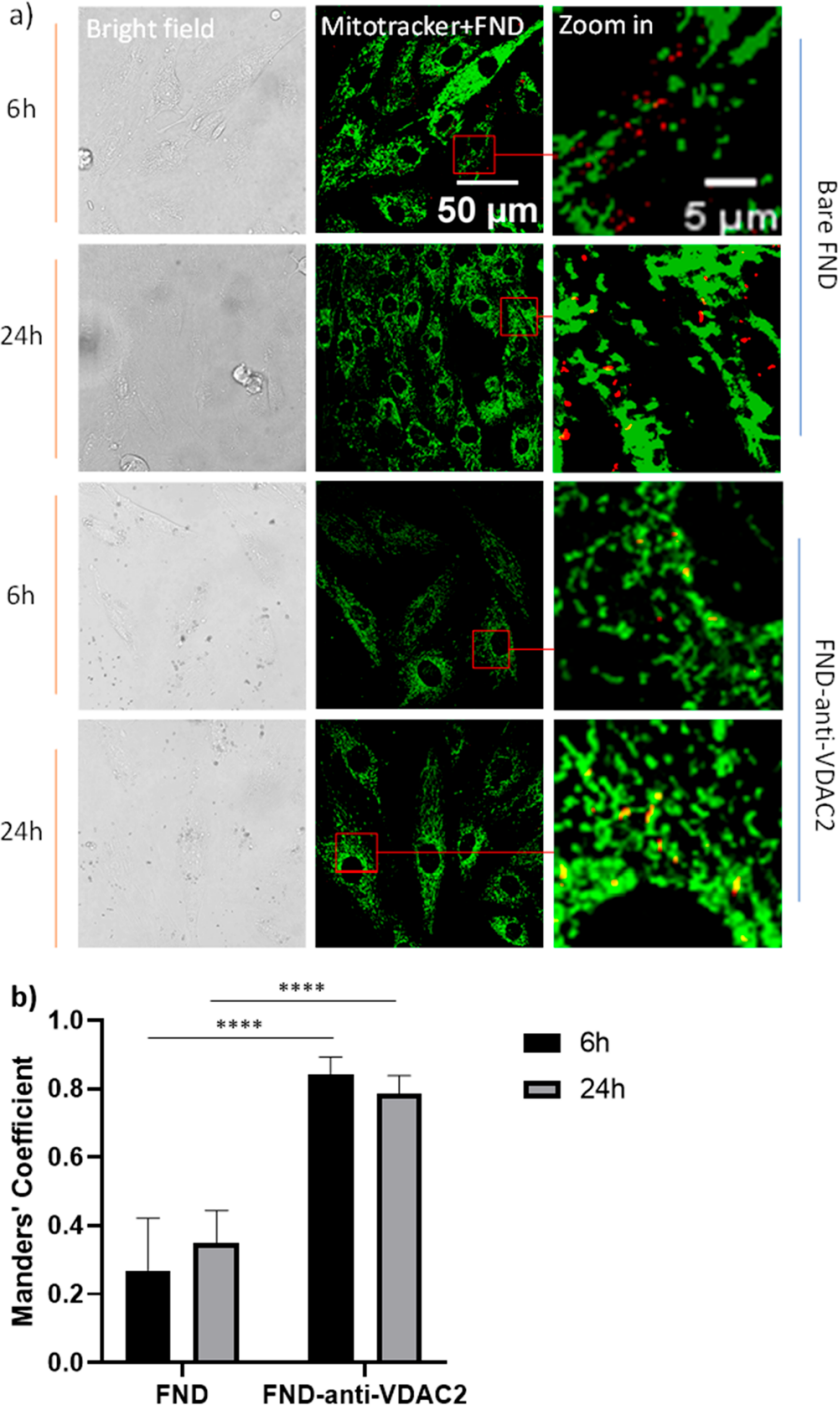
Subcellular locations of bare FNDs/FND-anti-VDAC2 and mitochondria were revealed by using an SP8x confocal microscope. (a) H9c2 cells were incubated with different FNDs for 6 or 24 h; then cells were stained and imaged. The location of mitochondria inside H9c2 cells was indicated by Mitotracker Green. Color code: green, Mitotracker; red, FNDs. (b) Quantitative colocalization analysis of bare FNDs/FND-anti-VDAC2 and mitochondria after incubating for 6 or 24 h. Error bars represent the standard deviations. The data were analyzed by two-way ANOVA. ****p ≤ 0.0001.

To measure the free radical production near mitochondria, it was necessary to ensure the presence of FNDs in the targeted region during relaxometry measurements. In this study, we examined whether diamond particles colocalized with mitochondria after a specific incubation period. As a control, the colocalization of bare FNDs with mitochondria was also assessed. The intracellular localization of particles was determined through confocal Z-stack imaging (Figure 6a). Mitochondria were labeled with Mitotracker Green, and the extent of colocalization between nanodiamond particles and mitochondria was quantified using Manders’ coefficients (MCs) (Figure 6b and Table 1), which are widely used for organelle colocalization analysis.^37,51,52^

**Table 1:** Manders’ Coefficient of Mitochondria and FND/FND-anti-VDAC2 after Different Incubation Times (from Figure 6b)^a^

	FND	FND-anti-VDAC2
6 h	0.27 ± 0.15	0.84 ± 0.05
24 h	0.35 ± 0.09	0.78 ± 0.05

a Error bars represent the standard deviations. The data were analyzed by two-way ANOVA.

Figure 6a shows a high degree of colocalization between FND-anti-VDAC2 particles and Mitotracker Green, indicating successful targeting of FNDs to the mitochondria after 6 and 24 h of incubation. In contrast, there are relatively fewer instances of colocalization between bare FNDs and mitochondria. These findings were further confirmed by image deconvolution and statistical analysis using FIJI and the JACoP plugin (Figure 6b and Table 1). Significant increases in the Manders coefficient were observed in the FND-anti-VDAC2 group compared to the bare FNDs at both 6 and 24 h of incubation. Through the colocalization analysis, we verified that we were indeed measuring free radical signals near mitochondria during the T1 measurement at the corresponding incubation time.

### Nanodiamond Biocompatibility

**Figure 7:**
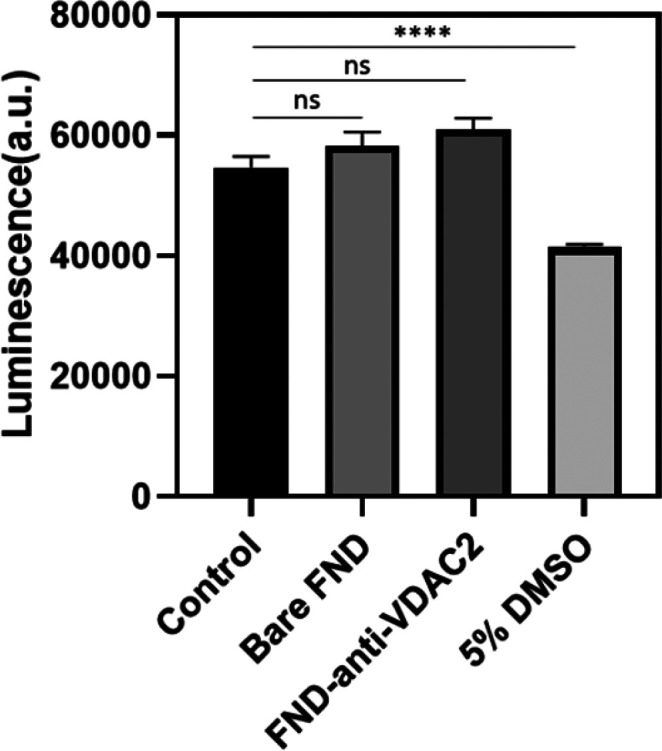
Cell viability was tested by a cell titer assay. Cells exposed to 5% DMSO were used as a positive control. Data are shown from three independent experiments. Error bars represent the standard deviations for each group. We determined significance between groups with one-way ANOVA. Statistical differences are indicated by ****p ≤ 0.001; ns = no significant difference.

To assess nanodiamond biocompatibility, we performed a cell titer assay (Figure 7) on H9c2 cells incubated with 1 μg/mL bare FNDs, 1 μg/mL FND-anti-VDAC2, or 5% dimethyl sulfoxide (DMSO) for 24 h. DMSO was used as a positive control due to its known toxicity, and the significant difference between the control and DMSO group indicated cells could be affected by toxic materials. We observed no statistically significant differences between cell viability of the control and the experimental groups where cells were exposed to different types of FNDs. These findings indicate that FNDs are well tolerated by H9C2 cells. These findings are consistent with previous reports of excellent biocompatibility of FNDs for different types of cells.^53−55^

### Reactive Oxygen Species Measurements

**Figure 8:**
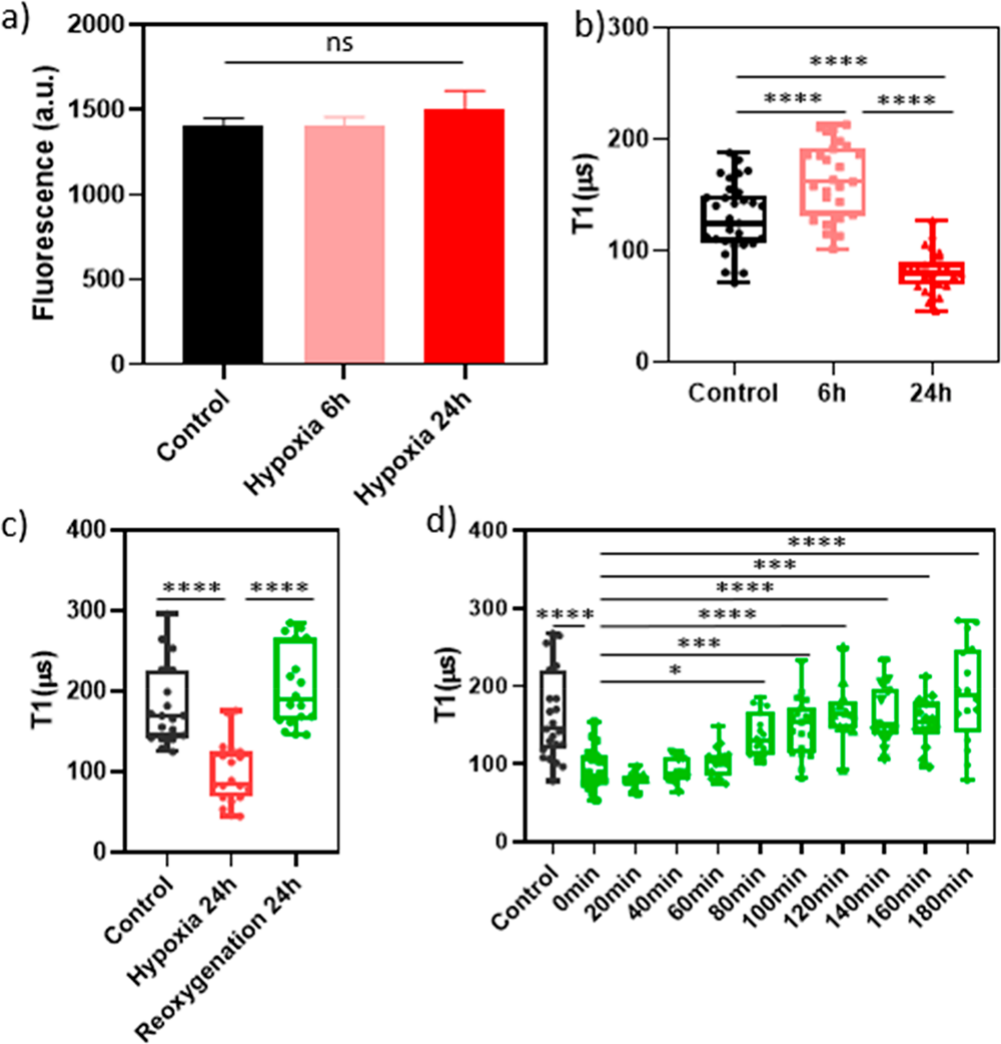
Free radical generation during normoxia/hypoxia/reoxygenation was determined by (a) a dihydroethidium (DHE) assay (detecting peroxide and superoxide) and T1 measurements of H9c2 cells (b) during 6 or 24 h hypoxia, (c) during 24 h hypoxia or 24 h reoxygenation, and (d) tracking free radical levels during 180 min reoxygenation after 24 h hypoxia. Whiskers represent min and max values, and data between each group in (a), (b), and (d) were analyzed by one-way ANOVA analysis; data between each group in (c) were analyzed by Student’s *t* test: ns = no significant difference, *p ≤ 0.1, ***p ≤ 0.001, ****p ≤ 0.0001.

The dihydroethidium (DHE) assay was used to compare the T1 data with conventional sensing in the bulk sample. DHE was taken up by the cells and underwent oxidation by O_2_^•–^, resulting in the production of ethidium, which binds to DNA and emits red fluorescence. This probe is commonly used to measure intracellular levels of superoxide and hydrogen peroxide.^56,57^ As the duration of hypoxia increased, the metabolic stress in the cells also increased (Figure 3). However, despite metabolic stress, the generation of reactive oxygen species was not detected by the DHE assay (Figure 8a). Since the mitochondrial electron transport chain generated reactive oxygen species (ROS) via electrons that leaked from ETC components and bound to oxygen to produce superoxide anions, mitochondrial ROS production was tightly linked to the availability of oxygen.^3^ Low oxygen availability (<1%) during hypoxia limited the production of superoxide, which limited the detection by the DHE assay.

In contrast, T1 relaxometry revealed significant differences in free radical levels compared to those in the DHE assay (Figure 8b), indicating that T1 measurements were more sensitive. While there are reports of single NV measurements,^58,59^ ensemble measurements are beneficial here. In ensemble measurements, each measurement reveals an average of all NV centers and is thus way more reproducible. Single NV centers on the other hand vary a lot, and it is thus impossible to compare measurements with different particles. Figure 8b shows a significant increase in T1 after 6 h of hypoxic treatment, indicating a notable decrease in the levels of free radicals near mitochondria. This is attributed to the limited production of free radicals under low oxygen conditions or cellular adaptation to hypoxia due to the stabilization and translocation of HIF1α, as demonstrated in Figure 6b. Moreover, the level of free radicals significantly increased after 24 h of hypoxia, suggesting cellular overload resulting from prolonged exposure to low oxygen levels, consistent with the findings in Figure 3b.

Interestingly, we also observed that the level of free radicals significantly decreased when hypoxic cells returned to normal cell culture conditions after 24 h of reoxygenation (Figure 8c). To gain a clearer understanding of when this decrease in free radicals occurred, we measured for 180 min during the later stages of the reoxygenation process, starting from the end of the hypoxia period (0 min). The results depicted in Figure 8d demonstrate that the T1 value progressively increased as the reoxygenation time extended, indicating a gradual reduction in free radical levels over time. Notably, a significant difference in T1 values was observed starting from 80 min and beyond, highlighting the diminishing presence of free radicals.

As controls, we measured the effect of the O_2_ and VDAC2 antibodies on the T1 measurement (Figures S1 and S2), and no measurable effects were observed. Additionally, T1 measurements using bare FNDs were conducted in cells under different environmental conditions (Figure S3), and no significant differences were found between the different groups, suggesting that most of the free radicals were produced near mitochondria.

### Study Limitations

The most severe limitation of this technique is that there needs to be a diamond particle at the location of the measurement, and measurements are limited to locations where nanodiamond particles go. In some cases, it is also difficult to know or control this location. In addition, since readout is optical, there needs to be an optical access. This means that one cannot perform measurements deep within thick samples or opaque samples. Finally, there is a variation between nanodiamonds, which limits the accuracy of relaxometry measurements.

## Conclusions

Here we have demonstrated that relaxometry can monitor the free radical load on the mitochondria surface when cells are stressed in a hypoxic environment. The changing trend of HIF1α at the same time indicates a possible link between ROS/free radicals, cell redox status, and hypoxic adaption. Compared to fluorescent assays, such as DHE, T1 measurements were performed continuously without the issue of fluorescence bleaching. While the DHE assay provided the history of the sample, T1 measurements provide real-time information. It is important to note that T1 measurements specifically represented local information from the surface of the mitochondria, confirming the occurrence of free radical generation on the mitochondria during hypoxia in H9c2 cells. Overall, relaxometry can be useful to determine the underlying mechanisms of oxidative stress response and hypoxic adaption.

## Materials and Methods

### Materials

The FNDs utilized in this study (Adamas Nanotechnologies in North Carolina, USA) had a hydrodynamic diameter of 70 nm. Additionally, they contain over 300 nitrogen-vacancy centers (NV^–^) per particle (according to the manufacturer).

They were synthesized using high-pressure high-temperature (HPHT) methods, followed by irradiation (with high-energy electrons at 3 MeV and a fluence of 5 × 10^19^ e/cm^2^) and high-temperature annealing.^31^ The manufacturer subjected the particles to a cleaning process using oxidizing acids. This process resulted in FNDs with oxygen groups on their surface, which were characterized in previous work.^32^ These FNDs are preferred over smaller particles for their brightness and ease of tracking, ensuring a favorable signal-to-noise ratio. The reason is that larger FNDs contain a higher number of NV centers, leading to more consistent signals.^26^ Each measurement represented an average of all of the NV centers, enhancing the reliability of the signals from the particles. Using even larger particles is also not ideal since in larger particles, NV centers would be too far away from the diamond surface. These FNDs demonstrated biocompatibility and maintained stable fluorescence after being taken up by cells.^33,34^

### Diamond Preparation

Antibody attachment has been previously established.^26^ In this study, anti-VDAC2 antibodies ([C2C3], C-term, catalog no. GTX104745) obtained from GeneTex (The Netherlands) were diluted to a concentration of 0.089 mg/mL (1:100 dilution as recommended by the manufacturer). To produce nanodiamonds that can be targeted to mitochondria, we mixed antibodies with 1 μg/mL FNDs (1:4 ratio) for 1 to 2 min by vortexing. Subsequently, the mixture was incubated at room temperature (RT) for 15 min to enable the antibodies to adsorb onto the FNDs, resulting in FND-anti-VDAC2. VDAC2 was proven to promote the endosome maturation process, which regulates the endocytosis pathway. This permits the escape of FNDs from endocytosis and thus enables mitochondria targeting.^35^ Also, these particles have been characterized before, and their ability to bind to mitochondria has been demonstrated.^26^ A Malvern ZetaSizer Nanosystem (dynamic light scattering; Malvern Instruments Ltd., Malvern, UK; www.malvern.com) was employed to assess any changes in size following the modification of the nanodiamonds.

### Cell Culture

In this article we used H9c2 embryonic rat heart-derived (ventricular) cells (myoblasts), which were purchased from ATCC. These cells were cultured in Dulbecco’s modified Eagle’s medium (DMEM), supplemented with 10% fetal bovine serum (FBS) and 1% penicillin/streptomycin. To maintain cells under physiological conditions, we kept them at 95% air/5% CO_2_. When the cells reached 50–60% confluency, they were subcultured.

### Cell Viability Test

To assess the cell viability, we employed a CellTiter-Glo luminescent cell viability assay (Promega). This assay measures ATP levels as indicators for metabolically active cells. H9c2 cells were seeded in clear flat-bottom 96-well plates at a density of 50,000 cells per well. To remove the remaining medium, which might cause aggregation of nanodiamonds, we discarded the cell culture medium and rinsed with phosphate-buffered saline (PBS). Subsequently, the cells were incubated with 1 μg/mL FNDs/FND-anti-VDAV2. As a positive control, we added 5% DMSO and kept the cells in DMSO for 24 h. After the incubation period, the plate and its contents were equilibrated to room temperature for ∼30 min. We added 100 μL of CellTiter-Glo 2.0 reagent to 100 μL of cells (in medium) followed by thoroughly mixing on an orbital shaker for 2 min to lyse cells. Ten minutes of incubation at room temperature concluded the staining process. Then we measured the luminescence with a FLUOstar Omega microplate reader (BMG Labtech, De Meern, The Netherlands). Untreated cells served as a negative control.

### FND Uptake in H9c2 Cells

To investigate FND uptake, cells were seeded (50,000 cells/mL) in 35 mm glass-bottom Petri dishes and incubated with bare FNDs or FND-anti-VDAC2 (1 μg/mL) in cell culture medium for 6 or 24 h. Cells were left in an incubator at 37 °C and 5% CO_2_. At the respective points when measurements were taken, the culture medium containing the FNDs was removed. Then cells were washed with 1× PBS. For staining, cells were fixed with 4% PFA for 10 min. To visualize the nuclei, we added DAPI, and fluorescein phalloidin (FITC-phalloidin) was added for staining F-actin (to visualize the cytoskeleton) and acquired Z-stack confocal images using a SP8× confocal microscope (Leica, Germany). FNDs were excited at the excitation maximum at 561 nm, and the emitted light was collected at 659 nm. DAPI and FITC were excited at 358 and 495 nm. The emission of the dyes was collected at 510 and 461 nm as suggested in previous literature.

For analysis, we selected around 60 cells randomly. Then Z-stack images of the entire cell volume of each cell were taken, and we outlined the cell regions for each cell. Subsequently, we quantified the number of FNDs per cell with FIJI (using the 3D object counter plugin). As size filter settings, we used 8 pixels and 26 as gray level threshold.

### Subcellular Location of FNDs in H9c2 Cells

To determine where diamond particles inside H9c2 cells end up after different incubation times, we labeled mitochondria with MitoTracker Green (Gibco, Thermo Fisher Scientific, The Netherlands). A total of 50,000 cells/mL were seeded in 35 mm glass-bottom Petri dishes. After attaching to the dish 1 μg/mL of bare FNDs or FND-anti-VDAC2 was added. These dishes were left for incubation for 6 or 24 h and then washed with PBS three times. After that, 1 μg/mL MitoTracker Green was added, and the cells were incubated for another 30 min. Sixty random living cells were then imaged using an SP8x Leica confocal microscope. For each experimental group, we performed three independent experiments. FNDs were detected at ex/em = 561/659 nm as above, while Mito Tracker Green was excited at 495 nm. The emission was measured at 510 nm. The resulting Z-stack images were further processed by deconvolution. To perform this step, we made use of Diffraction PSF 3D and iterative deconvolve 3D plugins of FIJI to improve image quality. To evaluate the intracellular location of FNDs, the JAcoP plugin^36^ in FIJI (https://imagej.nih.gov/ij/plugins/track/jacop.html) was used. More specifically, we analyzed whether FNDs colocalized with MitoTracker Green (mitochondria). The Manders coefficient, which was widely used for organelle colocalization analysis, indicated the fraction of FNDs in compartments containing mitochondria.^37^

### Hypoxia–Reoxygenation

H9c2 cells were exposed to varying durations of hypoxia for 6 or 24 h. A 6–24 h time range is widely used to establish H9c2 myoblast hypoxic models,^38−40^ and at 6 h, cells start to show the hypoxic features.^40,41^ We used an AnaeroPack system from Mitsubishi, Tokyo, Japan, to offer a hypoxic environment. This system is user-friendly, did not require the use of water or catalysts, and has been applied to some studies.^41,42^ The cells were placed in a 1 L anaerobic bag containing a sachet of carbon and a dry anaerobic indicator. Subsequently, they were incubated at 37 °C in a serum-free medium. Within ∼1 h, the oxygen concentration in the bag was expected to decrease to less than 1%. To conclude the hypoxic conditions, the anaerobic bag was opened and the cells were transferred to a normal oxygen environment (normoxia). Control cells were maintained at 37 °C in a standard culture incubator throughout the preparation process.

### Mitochondria Morphology

The cells were seeded (50,000 cells/mL) in 35 mm glass-bottom Petri dishes and incubated for different durations in a hypoxic environment; live cells were then stained by MitoTracker Green (1 μg/mL). After being incubated for 30 min, cells were imaged using an SP8x confocal microscope.

### HIF-1α Expression Level Measurement

To determine HIF-1α expression, cells were first seeded (50,000 cells/mL) in 35 mm glass-bottom Petri dishes. Then the cells were incubated for 6 or 24 h in a hypoxic environment. After that, cells were fixed with 4% PFA for 10 min. To assess the expression of HIF-1α, mouse HIF-1α monoclonal primary antibody (Abcam, UK) and goat anti-mouse-FITC secondary antibody (Abcam, UK) were used to stain HIF-1α. Nuclei were stained with DAPI. Z-stack confocal imaging was conducted using the same parameters as above.

To analyze the fluorescence intensity of HIF-1α in different regions of the cell using FIJI, the desired cell regions were outlined using the Freehand ROI tool. Area, integrated density, and mean gray value were checked. Background fluorescence was read from the control cell group and corrected when calculating the final mean optical intensity.

### Measuring Free Radical Generation in H9c2 Cells by T1 Measurement

A previously described home-built magnetometry setup was used for T1 measurements.^43^ H9c2 cells were seeded in 35 mm glass-bottom Petri dishes and incubated overnight. To study free radical generation near mitochondria during hypoxia, H9c2 cells were cultured with 1 μg/mL of FND-anti-VDAC2 for 6 or 24 h in a hypoxic environment. To remove surplus diamond particles that were not internalized, we washed the samples with PBS and added a serum-free culture medium. T1 measurements were performed immediately and repeated three times for each group. Normoxic cells were used as controls for the T1 measurements. For reoxygenation, H9c2 cells were cultured with 1 μg/mL of FND-anti-VDAC2 for 24 h in a hypoxic environment. After washing out the free particles with PBS and replacing them with a complete cell culture medium, T1 measurements were performed every 20 min in the same sample, repeated three times, to track free radical generation.

During T1 relaxometry measurements, we utilized NV defects in nanodiamonds. These defects enable quantum sensing at room temperature. This means that they read out the magnetic noise of the surroundings optically.^32^ To perform these measurements, we used a confocal setup, which allows laser pulsing and has been described earlier.^25^ To perform a typical relaxometry experiment, we utilized the pulse sequence in Figure 1a.

To this end, NV centers are brought in the bright ms = 0 state with a laser pulse, and then we probe after different dark times if the NV centers are still in the bright state or returned to the thermal equilibrium between ms = 0 and the darker ms = +1 and −1 state. In such a measurement, the time it takes the NV centers to return to the darker equilibrium state is reduced in the presence of magnetic noise (from free radicals in this case).

For pulsing the NV centers were excited with 5 μs green laser pulses (561 nm, 50 μW at the position of the sample). These pulses were interrupted by dark times (τ) from 200 ns to 10 ms. To generate relaxometry curves, we plotted the brightness in the first 0.6 μs of each pulse against the dark time (shown in Figure 1b). From these curves we calculate T1 using a biexponential model (equation shown in Figure 1b), which is further explained in previous work.^44,45^ For a better signal-to-noise ratio, the pulsing sequence was repeated 10,000 times per measurement.

### Dihydroethidium Assay

A total of 50,000 H9c2 cells were seeded per 96-well plate with a clear flat bottom. Following hypoxia treatment for either 6 or 24 h, we washed the cells with PBS. We replaced PBS by 200 μL of a solution of DHE (2 μg/mL) in DMEM. The cells were subsequently incubated for 10 min at 37 °C and 5% CO_2_. DHE is a fluorescent probe specifically used for detecting the generation of ROS, including, for instance, superoxide and hydrogen peroxide. To measure ROS, we detected the green fluorescence (580 nm, excited at 514 nm) using a FLUOstar Omega microplate reader (BMG Labtech, De Meern, The Netherlands). Cells incubated under normoxic conditions were used as negative controls. The experimental procedure followed the instructions provided in the manufacturer’s manual.

### Statistical Analysis

Statistical analysis of the data was performed using GraphPad Prism version 8.0. The significance of the results was determined using either a one-way or two-way ANOVA test (Tukey multiple comparisons), depending on the specific experiment. Significance was assessed by comparing the experimental groups to the control group, and the significance levels were defined as follows: ns (not significant) for *p* > 0.05, * for *p* ≤ 0.05, ** for *p* ≤ 0.01, *** for *p* ≤ 0.001, and **** for *p* ≤ 0.0001.

## Supporting Information Available

Control experiment for T1 measurements with FNDs exposed to hypoxia and normoxia; control experiments with bare FNDs and FND-anti-VDAC2; control measurements with nontargeted FNDs; demonstration of photostability of FNDs; viability test of cells before and after laser exposure (PDF)
