# Supplementary material for: Quantum Sensing of Free Radical Generation in Mitochondria of Single Heart Muscle Cells during Hypoxia and Reoxygenation
Source: ACS Nano. 2024 Jan 18;18(4):2982–91. doi: 10.1021/acsnano.3c07959 (PMC10832053; doi:10.1021/acsnano.3c07959)
Supplement: Supplementary file 1 — nn3c07959_si_001.pdf [file nn3c07959_si_001.pdf]

# Supporting information

### Quantum sensing of free radical generation in mitochondria of single heart muscle cells during hypoxia and reoxygenation

Siyu Fan[1], Han Gao[1,2], Yue Zhang[1], Linyan Nie[1], Raquel Bártolo[1], Reinier. Bron[1], Hélder A. Santos[1,2], Romana Schirhagl[1]

[1] Department of Biomedical Engineering, W.J. Kolff Institute for Biomedical Engineering and Materials Science, University Medical Center Groningen, University Groningen, Antonius Deusinglaan 1, 9713 AV Groningen, The Netherlands

[2] Drug Research Program, Division of Pharmaceutical Chemistry and Technology, Faculty of Pharmacy, University of Helsinki, Helsinki, Finland

*Corresponding author. Email: romana.schirhagl@gmail.com

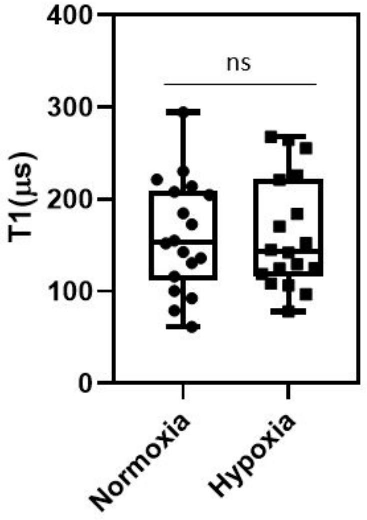


*Figure S1 To see the effect of $O_2$ to T1 magnetometry, Bare FND was used to do T1 measurement in normoxia and hypoxia-treated medium, respectively. Whiskers represent min and max values. Data between each group were analyzed by t-test analysis: ns=no significant difference.*

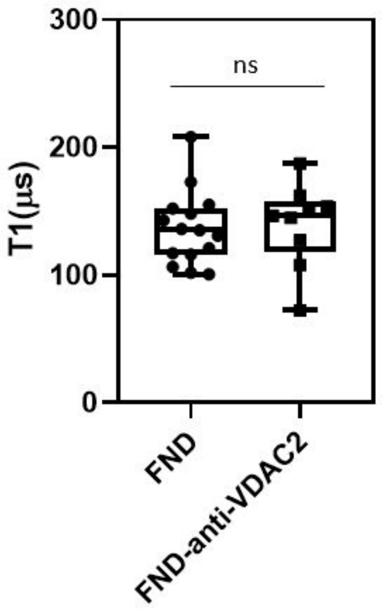


*Figure S2 T1 measurement of bare FND and FND-anti-VDAC2. Data between each group were analyzed by t-test analysis: ns=no significant difference.*

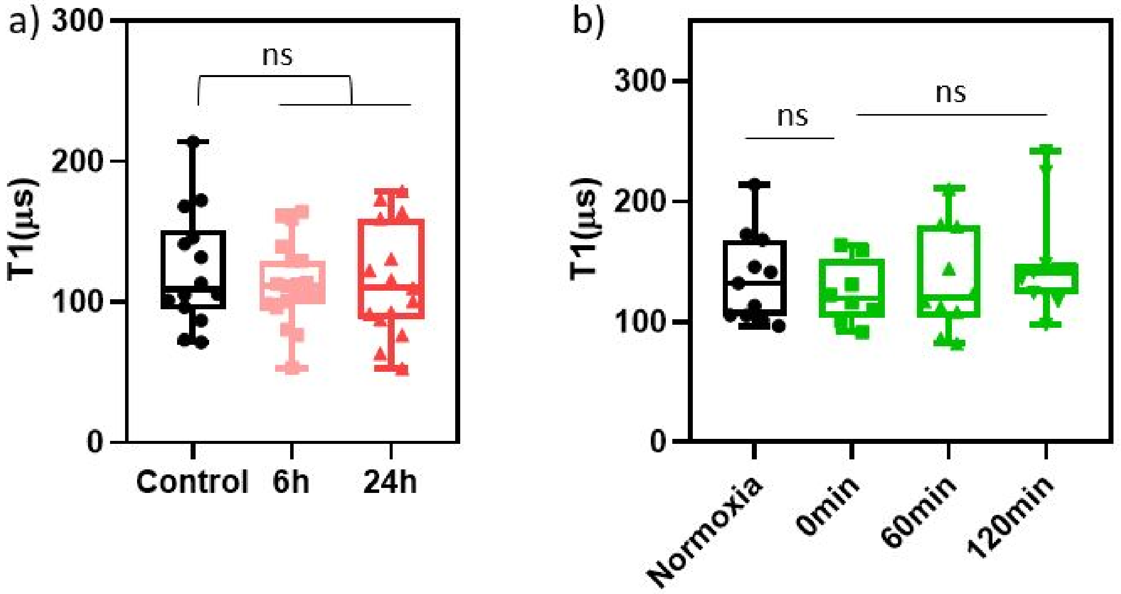


*Figure S3 Non-targeted bare FNDs were used to do T1 measurement in H9c2 cells under a) different hypoxic conditions and b) different reoxygenation conditions. Data between each group were analyzed by one-way-anova analysis: ns=no significant difference.*

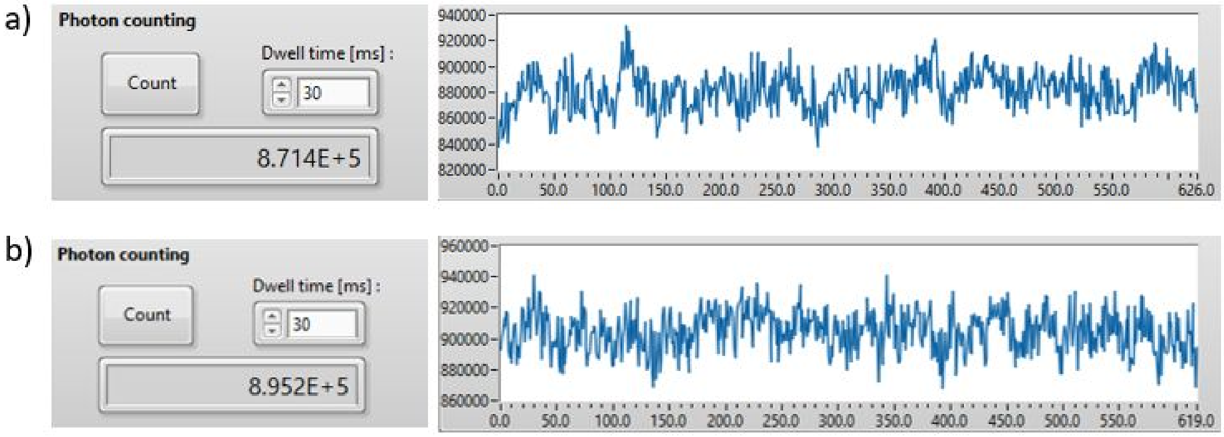


*Figure S4 Representative photostability test of a single nanodiamond . x axis: time (s/100), y axis: The counted photons. a) Before T1 measurement; b) after 3h T1 measurement. The recorded photon numbers remained stable within the tracking window. Also, after 3h of T1 measurement, the photon numbers didn't decrease, which indicated the photostability of nanodiamonds.*

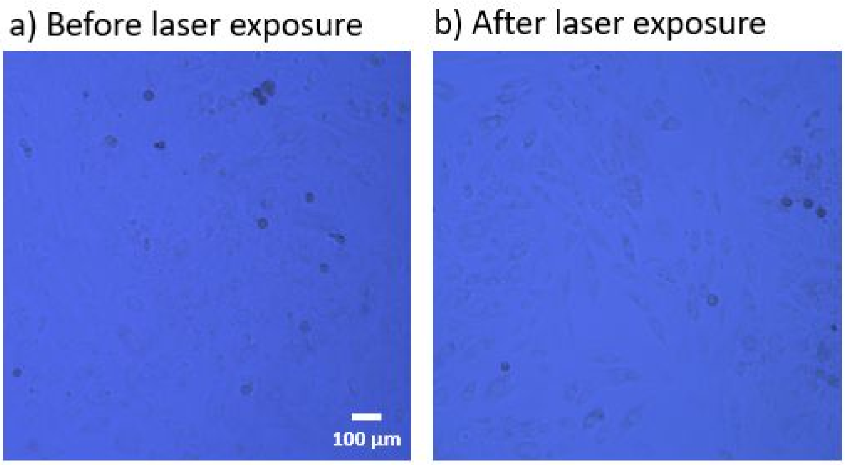


*Figure S5 Images of 1% trypan blue stained H9c2 cells a) before and b) after the 50 µW laser exposure over a period of 3 hours. No significant cell death was observed in the groups after laser exposure.*
